# Open kyphoplasty in the treatment of a painful vertebral lytic lesion with spinal cord compression caused by multiple myeloma: A case report

**DOI:** 10.3892/ol.2013.1222

**Published:** 2013-03-01

**Authors:** JUN PAN, ZHONG-LAI QIAN, ZHI-YONG SUN, HUI-LIN YANG

**Affiliations:** Department of Orthopaedic Surgery, The First Affiliated Hospital of Soochow University, Suzhou, Jiangsu 215006, P.R. China

**Keywords:** spinal cord compression, vertebral lytic lesion, kyphoplasty, pedicle screw fixation

## Abstract

Multiple myeloma is a fatal hematological malignancy, with the most common localization being the spine. A 72-year-old male patient presented with progressive back pain and dysfunction of ambulation. Spinal computed tomography (CT) and magnetic resonance imaging (MRI) showed spinal cord compression at the T9-T10 level due to an extensive epidural mass in the spinal canal, a large lytic mass of T7-T12 with extraosseous extension and involvement of T9 and T10 vertebral pedicle and posterior wall. The patient underwent posterior spinal decompression and kyphoplasty of T9 and T10 with pedicle screw fixation in T7, T8, T11 and T12. Pain and neural function were improved significantly postoperatively. To our knowledge, such methods have rarely been used to treat a patient with intractable back pain and neurological compromise with multiple myeloma or spinal metastases.

## Introduction

Multiple myeloma is a B-cell disorder characterized by accumulation of malignant plasma cells, generally derived from one clone in the bone marrow (1). It accounts for ∼1% of all malignant diseases and represents ∼10% of hematologic malignancies (2). The intricate interactions between an increase in osteoclastic bone resorption and a reduction in bone formation usually cause bone destruction, with the most common localization being the spine. The condition is associated with severe bone pain, pathological fractures, osteoporosis and spinal cord compression (3). Spinal cord compression occurs in ∼5% of patients with multiple myeloma (4). In the present study, a case of multiple myeloma with a large, lytic bone of the vertebral body and spinal cord compression is described, which was treated by laminectomy, pedicle screw fixation and kyphoplasty, known as open kyphoplasty (OKP). To our knowledge, such methods have rarely been used to treat a patient with intractable back pain and neurological compromise resulting from multiple myeloma or spinal metastases.

## Case report

A 72-year-old male was referred with the complaint of severe back pain and dysfunction of ambulation. The back pain had begun two months prior to admission and was preceeded by a history of weakness and significant weight loss. The patient was unable to walk due to progressive back pain and heaviness of both lower extremities. Physical examination showed tenderness in the T9-T10 region, hypertension of both lower extremities without paraparesis, sensory loss, sphincter disorder or abnormal reflexes. The study was approved by the Ethics Committee of The First Affiliated Hospital of Soochow University, Suzhou, China. Written informed consent was obtained from the patient.

**Figure 1:**
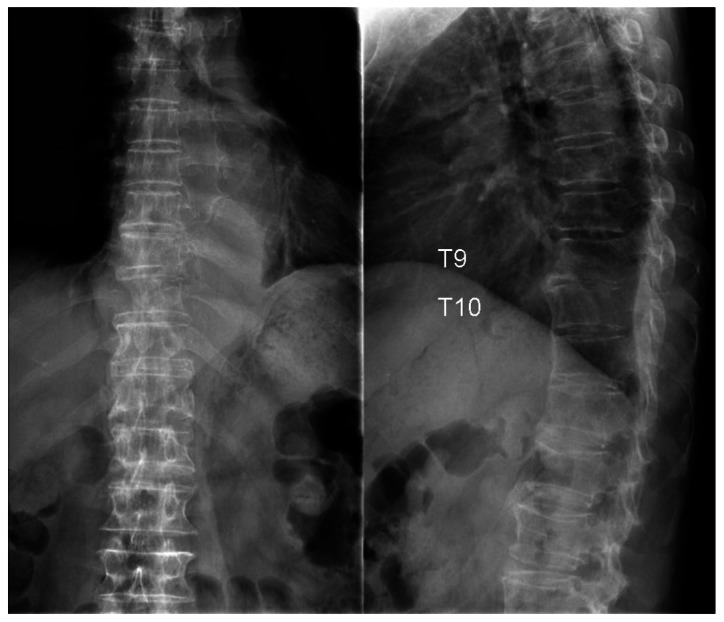
The AP and lateral X-ray showed lytic involvement of T9 and T10 vertebral pedicles and posterior wall.

**Figure 2:**
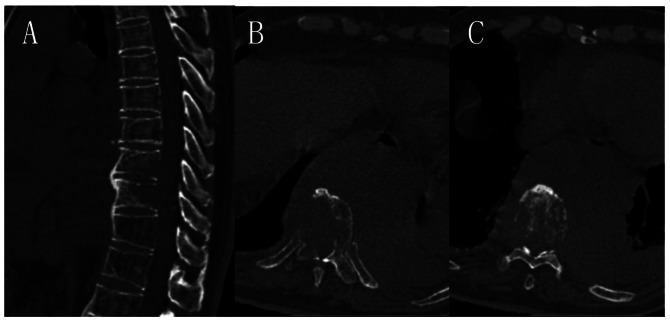
The three-dimensional computed tomography (CT) from T5 to T12. (A) The posterior wall of T9 and T10 was eroded by the tumor mass on sagittal images. (B-C) The CT scans showed extensive lytic involvement of vertebral column and pedicles in T9 and T10, respectively.

**Figure 3:**
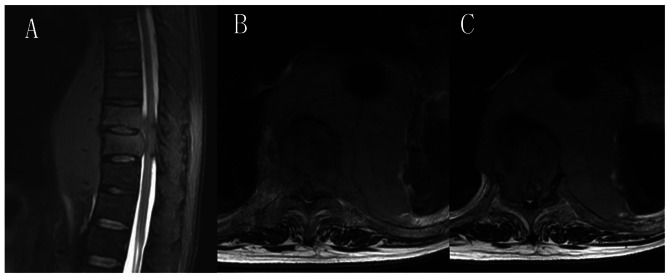
(A) The sagittal T2-weighted magnetic resonance imaging (MRI) showed a large lytic mass of the T7-T12 region with extraosseous extension. (B-C) MRI showed extradural spinal compression by a tumor mass (T9 and T10) with tumoral involvement of the entire bone marrow in T9 and T10, respectively.

X-ray, computed tomography (CT) and magnetic resonance imaging (MRI) of the thoracic spine revealed spinal cord compression at the T9-T10 level due to an extensive epidural mass in the spinal canal (Figs. 1–3). There was also a large lytic mass at the T7-T12 level with the extraosseous extension surrounding the abdominal aorta, and lytic involvement of T9 and T10. Initial laboratory studies revealed a Bence-Jones proteinuria and an erythrocyte sedimentation rate of 100 mm/h. Bone marrow aspiration of the posterior iliac crest showed an infiltration of atypical plasma cells.

Surgery was performed under general anesthesia, with the patient placed in the extended prone position, with padding beneath the upper chest and pelvic regions. The first operative phase involved osteosynthesis where 8 pedicle screws were placed at T7, T8, T11 and T12. Laminectomy of T9 and T10 was performed to achieve decompression of the spinal cord. This was followed by biopsy. The second phase of surgery involved kyphoplasty. An 11-gauge Jamshidi needle was placed into the posterior part of T9 via the left transpedicular approach as the right pedicle was totally eroded. The kyphoplasty systems (Kyphon, Sunnyvale, CA, USA) were placed into the T9 vertebral body through the left working channel. The balloon was inflated to 2 ml under fluoroscopic guidance until manometric parameters reached 150 Pa. Polymethyl methacrylate (PMMA) cement (2.5 ml) was placed into the cavity under continuous fluoroscopic monitoring in the lateral plane following the withdrawal of the balloon. The same procedure was performed in T10. The whole duration of the surgical intervention was 3.5 h and 400 ml red blood cells was transfused.

**Figure 4:**
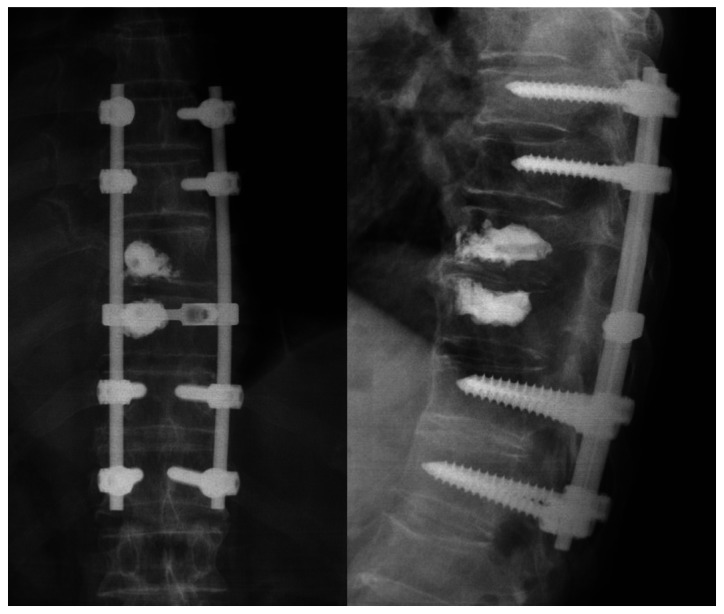
The AP and lateral X-ray postoperatively showed no cement leakage or mislocation of screws.

The patient tolerated surgery and showed a good clinical outcome. The day after the procedure, the patient had excellent alleviation of back pain without painkillers and the visual analogue scale (VAS) score was decreased from 8 to 2 points. Three days after surgery, the patient could ambulate with assistance. Two weeks after the operation, the patient was transferred to the Hematological Department for further chemotherapy and radiotherapy. The postoperative radiographs showed no cement leakage or mislocation of screws (Fig. 4). Histopathological examination of the tumor tissue confirmed multiple myeloma consistent with bone marrow aspiration.

## Discussion

Multiple myeloma is a fatal hematological malignancy associated with clonal expansion of malignant plasma cells within the bone marrow and the development of a destructive osteolytic bone disease (3). The median age at diagnosis is 68 years old and males are more frequently affected than females. Although chemotherapy and radiotherapy as noninvasive treatment have a major role in the management of multiple myeloma, they may have adverse effects on a patient’s immune system (5). Furthermore, neither of these treatment approaches protect the spine from progressive osteolytic collapse and spinal cord compression, which cause intractable pain, neurological compromise and overt or impending spinal instability. An effective alternative therapy is therefore required.

Posterior decompression and pedicle screw instrumentation supplemented with kyphoplasty, known as OKP, is recognized as an appropriate surgery to achieve pain relief, neurological improvement and spinal stability. OKP is not a new method. It was first reported by Hsiang (6) in 2003 to treat an osteoporotic vertebral compression fracture with fractured posterior cortex. Fuentes *et al*(7) recently reported the use of OKP in a series of 16 patients with severe osteoporotic compression fractures associated with neurological disorders, all of whom gained significant pain reduction and neurological improvement. Furthermore, Marco *et al*(8) used OKP with calcium phosphate instead of PMMA to treat 38 relatively young and healthy patients suffering from unstable thoracolumbar burst fractures with or without neurological deficit. They demonstrated that this method reconstructed and stabilized the anterior column, restored vertebral body height, indirectly and directly decompressed the thecal sac, reduced the kyphotic deformity and stabilized the posterior column, using a posterior approach. Open vertebroplasty (OVP) was recently described by Weitao *et al*(9), who reported that this method was used to treat 18 cases with spinal metastatic disease. Excellent pain relief and neural function recovery were obtained, apart from in 1 case where cement leakage into the pulmonary veins occurred due to the use of low viscosity cement and a high application pressure. To our knowledge, no study has evaluated the clinical outcome for patients with multiple myeloma with neurological deficits who have been managed with OKP.

With the development of minimally invasive surgery, vertebral augmentation has widely been used for intractable painful pathological vertebral fracture caused by multiple myeloma. Yang *et al*(10) reported that vertebroplasty combined with chemotherapy in the treatment of multiple myeloma-associated spinal fracture showed significant improvement of pain relief. Kyphoplasty as a modified version of vertebroplasty involved inflation of a balloon within a collapsed vertebral body to allow a void injection of PMMA. A report from Zou *et al*(11) involved 21 myeloma patients with vertebral compressive fractures who underwent 43 kyphoplasty procedures which provided a significant and sustained reduction of pain, resulting in a significant functional improvement for the multiple myeloma patients. Several analgesic and antitumor mechanisms of PMMA were proposed, including stabilization of vertebral microfracture and enhancement of bone support force, both monomer cytotoxicity and thermal effect on tumor cell and pain nerve endings, and blood supply cut off by solidification of cement (12,13). In the present study, we described a case of back pain of VAS 8 points which was reduced to 2 points immediately after surgery. The effect has lasted to the latest follow-up without additional painkillers.

Both vertebroplasty and kyphoplasty have been shown to substantially reduce pain from vertebral collapse caused by myeloma but have the same complication of cement leakage into the spinal canal, neural foramina or pulmonary venous system. Moreover, the incidence of cement extravasation with kyphoplasty or vertebroplasty for myeloma is much higher than that associated with osteoporotic fractures due to cortical destruction and the enriched blood supply of myeloma (9). Lee *et al* used a meta-analysis and reported that the rate of symptomatic cement leakage was 10% in metastatic disease or myeloma and only 1% in osteoporotic collapse (14). Furthermore, the rate in vertebroplasty is much higher than that in kyphoplasty. The largest North American series reporting augmentation of cement for metastatic spinal disease showed that leakage of cement occurred during vertebroplasty at six of 65 levels (9.2%) while no extravasation (0/32) was seen during kyphoplasty (15).

The large eroded vertebral posterior wall of T9 and T10 implied high risks of cement leakage and secondary neurological deterioration in the present case, which presented the greatest challenge of the procedure. Spinal canal compromise and disruption of the posterior cortex of the vertebral body have been considered as relative contraindications. In our study, to minimize the disruption of the posterior wall, continuous fluoroscopic monitoring was performed throughout the bone cement-filling process. The filling process was stopped as soon as the bone cement reached one-fourth of the distance to the posterior wall of the vertebrae (16). Unipedicular kyphoplasty was performed as the right side of vertebral pedicle and posterior wall were totally eroded. It was observed by La Maida *et al*(17) that unipedicular kyphoplasty demonstrate results comparable with those of bipedicular kyphoplasty in the treatment of multiple myeloma. In this case, no cement leakage into the spinal canal, neural foramina or venous system was found at X-ray, either postoperatively or by fluoroscopic monitoring during surgery (Fig. 4).

Vertebral augmentation has limitations in relieving spinal cord compression and stabilizing the spinal column, however. A surgical approach including laminectomy and pedicle screw fixation is therefore necessary. It was recognized that surgical decompression when performed without instrumentation, whether via a ventral or dorsal approach, caused further instability to the metastatic spine (18). In this case, decompression and osteosynthesis were performed ahead of kyphoplasty for several reasons. Firstly, the rate of spinal cord injury caused by the mechanically inflated balloon during kyphoplasty could be decreased significantly when the canal was decompressed. Secondly, laminectomy and decompression allowed direct visualization of the posterior vertebral wall for safe cement-filling and removal of cement leakage as soon as it was observed under fluoroscopic monitoring (15). In addition, the use of PMMA cement augmentation helped secure the pedicle screws when pathological fractures or kyphosis developed due to operative instability such as loss of posterior spinal elements (19,20).

OKP is a reasonable palliative surgery to treat multiple myeloma or spinal metastatic disease accompanied by spinal cord compression. It allows simultaneous decompression of the spinal cord and stabilization of the vertebral column in the same procedure and demonstrates excellent clinical results in pain relief and the recovery of neural function with less blood loss, shorter operation time and fewer complications.
